# Inhibition of IκB kinase reduces the multiple organ dysfunction caused by sepsis in the mouse

**DOI:** 10.1242/dmm.012435

**Published:** 2013-05-02

**Authors:** Sina M. Coldewey, Mara Rogazzo, Massimo Collino, Nimesh S. A. Patel, Christoph Thiemermann

**Affiliations:** 1 Queen Mary University of London, Barts and The London School of Medicine and Dentistry, The William Harvey Research Institute, London, EC1M 6BQ, UK; 2 Hannover Medical School, Department of Anesthesia and Intensive Care Medicine, 30625 Hannover, Germany; 3 University of Turin, Department of Drug Science and Technology, 10125 Turin, Italy

## Abstract

Nuclear factor κB (NF-κB) plays a pivotal role in sepsis. Activation of NF-κB is initiated by the signal-induced ubiquitylation and subsequent degradation of inhibitors of kappa B (IκBs) primarily via activation of the IκB kinase (IKK). This study was designed to investigate the effects of IKK inhibition on sepsis-associated multiple organ dysfunction and/or injury (MOD) and to elucidate underlying signaling mechanisms in two different *in vivo* models: male C57BL/6 mice were subjected to either bacterial cell wall components [lipopolysaccharide and peptidoglycan (LPS/PepG)] or underwent cecal ligation and puncture (CLP) to induce sepsis-associated MOD. At 1 hour after LPS/PepG or CLP, mice were treated with the IKK inhibitor IKK 16 (1 mg/kg body weight). At 24 hours, parameters of organ dysfunction and/or injury were assessed in both models. Mice developed a significant impairment in systolic contractility (echocardiography), and significant increases in serum creatinine, serum alanine aminotransferase and lung myeloperoxidase activity, thus indicating cardiac dysfunction, renal dysfunction, hepatocellular injury and lung inflammation, respectively. Treatment with IKK 16 attenuated the impairment in systolic contractility, renal dysfunction, hepatocellular injury and lung inflammation in LPS/PepG-induced MOD and in polymicrobial sepsis. Compared with mice that were injected with LPS/PepG or underwent CLP, immunoblot analyses of heart and liver tissues from mice that were injected with LPS/PepG or underwent CLP and were also treated with IKK 16 revealed: (1) significant attenuation of the increased phosphorylation of IκBα; (2) significant attenuation of the increased nuclear translocation of the NF-κB subunit p65; (3) significant attenuation of the increase in inducible nitric oxide synthase (iNOS) expression; and (4) a significant increase in the phosphorylation of Akt and endothelial nitric oxide synthase (eNOS). Here, we report for the first time that delayed IKK inhibition reduces MOD in experimental sepsis. We suggest that this protective effect is (at least in part) attributable to inhibition of inflammation through NF-κB, the subsequent decrease in iNOS expression and the activation of the Akt-eNOS survival pathway.

## INTRODUCTION

Sepsis is a complex clinical entity caused by an individual’s systemic response to an infection and has a wide range of clinical symptoms often leading to multiple organ dysfunction and/or injury (MOD) and ultimately multiple organ failure (severe sepsis). Sepsis-induced hypotension despite adequate fluid resuscitation is termed ‘septic shock’ (Bone et al., 1992). Severe sepsis and septic shock remain the leading causes of death in the non-coronary intensive care units and places a large burden on healthcare resources (Angus et al., 2001; Dombrovskiy et al., 2007; Wang et al., 2007). Despite substantial advances in our knowledge of the pathophysiology of sepsis, the treatment of this condition is still a clinical challenge. To date, therapies are mostly supportive in nature and all specific experimental therapeutic approaches, except early administration of antibiotics and ‘early goal-directed therapy’ (Rivers et al., 2001), have failed to be translated successfully into the clinical setting. Thus, new pharmacological strategies are urgently needed to improve the treatment of this condition.

There is now good evidence that a large number of interventions that inhibit the activation of NF-κB (nuclear factor κ-light-chain-enhancer of activated B cells) also reduce the MOD associated with sepsis (including septic cardiac and renal dysfunction). These interventions include treatment with: calpain-inhibitor-I (Ruetten and Thiemermann, 1997), ligands of peroxisome proliferator-activated receptor (PPAR)-β/δ (Kapoor et al., 2010; Zingarelli et al., 2010) or PPAR-γ (Abdelrahman et al., 2005; Zingarelli and Cook, 2005), insulin and other inhibitors of glycogen synthase kinase-3β (Dugo et al., 2006), and erythropoietin (Coldewey et al., 2013; de Souza et al., 2012; Khan et al., 2013), to name but a few. The NF-κB protein complex controls DNA transcription for a multitude of pro-inflammatory and immunological molecules (Moynagh, 2005; Sen and Baltimore, 1986). Inhibitors of κB (IκBs) mask the nuclear localization signals of NF-κB proteins and sequester NF-κB as an inactive complex in the cytoplasm (Jacobs and Harrison, 1998; Senftleben and Karin, 2002). Signal-induced ubiquitylation and subsequent proteolytic degradation of IκBs that have been phosphorylated by IκB kinase (IKK) liberate NF-κB to enter the nucleus and activate NF-κB target genes (Senftleben and Karin, 2002). The IKK complex consists of three distinct subunits, the catalytic subunits IKKα (IKK1) and IKKβ (IKK2) as well as the regulatory subunit IKKγ (NEMO) (Li et al., 1999b). However, there is great evidence that IKKβ is crucial for NF-κB activity and liver development in mice: IKKβ-deficient mice die at midgestation from uncontrolled liver apoptosis. IKKα can only partially compensate for the loss of IKKβ (Li et al., 1999a; Li et al., 1999b).

**TRANSLATIONAL IMPACT**

**Clinical issue**

Sepsis is the systemic inflammatory response to infection. The condition is associated with a wide range of serious clinical problems that can ultimately lead to multiple organ failure and death. Interventions that target specific aspects of sepsis pathophysiology have been successful in animal studies, but not in clinical trials. One obstacle to clinical translation is the high degree of variability in patient immune responses, necessitating the development of targeted therapies to treat stratified patient populations. There is mounting evidence that inhibition of NF-κB activation can reduce sepsis-associated organ dysfunction and injury. However, most of the inhibitors involved have to be administered too early to be useful in a clinical setting, underlining the need to develop interventions that target a later stage in NF-κB activation. The IκB kinase (IKK) complex is involved in one of the final steps in the pathway; therefore, inhibition of this complex could represent a promising approach for the timely control of sepsis-associated organ failure.

**Results**

Here, the effects of delayed administration of a specific IKK inhibitor, IKK 16, on multiple organ dysfunction and injury were examined in two different mouse models of experimental sepsis. The authors report that activation of the NF-κB protein complex is associated with organ dysfunction and injury in experimental sepsis caused by either excessive inflammation via co-administration of lipopolysaccharide and peptidoglycan (LPS/PepG) in young mice, or polymicrobial sepsis after cecal ligation and puncture (CLP) in aged mice. They demonstrate that delayed administration (1 hour after LPS/PepG or CLP) of IKK 16 ameliorates sepsis-induced multiple organ dysfunction and injury at 24 hours. Furthermore, their results are consistent with the protective effect being at least partly attributable to inhibition of NF-κB-mediated inflammation and the associated decreases in inducible nitric oxide synthase (iNOS) expression. Interestingly, a role for the well-known Akt-eNOS (endothelial nitric oxide synthase) survival pathway was also revealed.

**Implications and future directions**

This work provides convincing evidence that selective inhibition of IKK could be an effective strategy for the prevention or treatment of organ dysfunction and injury associated with sepsis and other conditions involving systemic inflammation. Future studies should seek to determine how late after the onset of sepsis an IKK-inhibitor can be administered in order to improve survival (e.g. mortality studies), and to find out whether inhibition of IKK has an impact on the host response. Ultimately, the efficacy of IKK 16 and similar molecules needs to be evaluated in large mammals before moving to Phase 1 safety studies in humans.

Owing to its rapid activation and unique regulation, the NF-κB pathway plays a pivotal role in the onset of sepsis. This raises the important, but hitherto unaddressed, question as to whether one can improve the MOD caused by sepsis by specifically inhibiting IKK.

Thus, this study was designed to investigate the role of the delayed administration of a specific IKK inhibitor, IKK 16 (Waelchli et al., 2006), in severe lipopolysaccharide and peptidoglycan (LPS/PepG)-induced MOD and in a model of cecal ligation and puncture (CLP)-induced polymicrobial sepsis *in vivo*.

Specifically, we have investigated: (1) the response of C57BL/6 mice to LPS/PepG (young mice; end points: cardiac and renal dysfunction, hepatic injury, lung inflammation); (2) the effects of IKK 16 on this response; (3) the effects of IKK 16 in the more clinically relevant model of CLP-induced polymicrobial sepsis with fluid resuscitation and antibiotic therapy (aged mice; end points: cardiac and renal dysfunction, hepatic injury, lung inflammation); and (4) the signaling events underlying the observed beneficial effects of IKK 16 in hearts and livers of mice after co-administration of LPS/PepG or CLP, including the degree of IκBα phosphorylation, the nuclear translocation of NF-κB subunit p65, the tissue levels of inducible nitric oxide synthase (iNOS), and the degree of phosphorylation of Akt and endothelial nitric oxide synthase (eNOS), all of which are known to mediate some of the key effects in experimental sepsis in many organs, including the heart and the liver.

Both animal models employed in this study recapitulate some individual features of sepsis while minimizing others and therefore complement each other to enable us to gain a better and more complete insight into the role of IKK inhibition in sepsis.

## RESULTS

### Effect of LPS/PepG administration and treatment with IKK 16 on cardiac function in mice

**Fig. 1.**
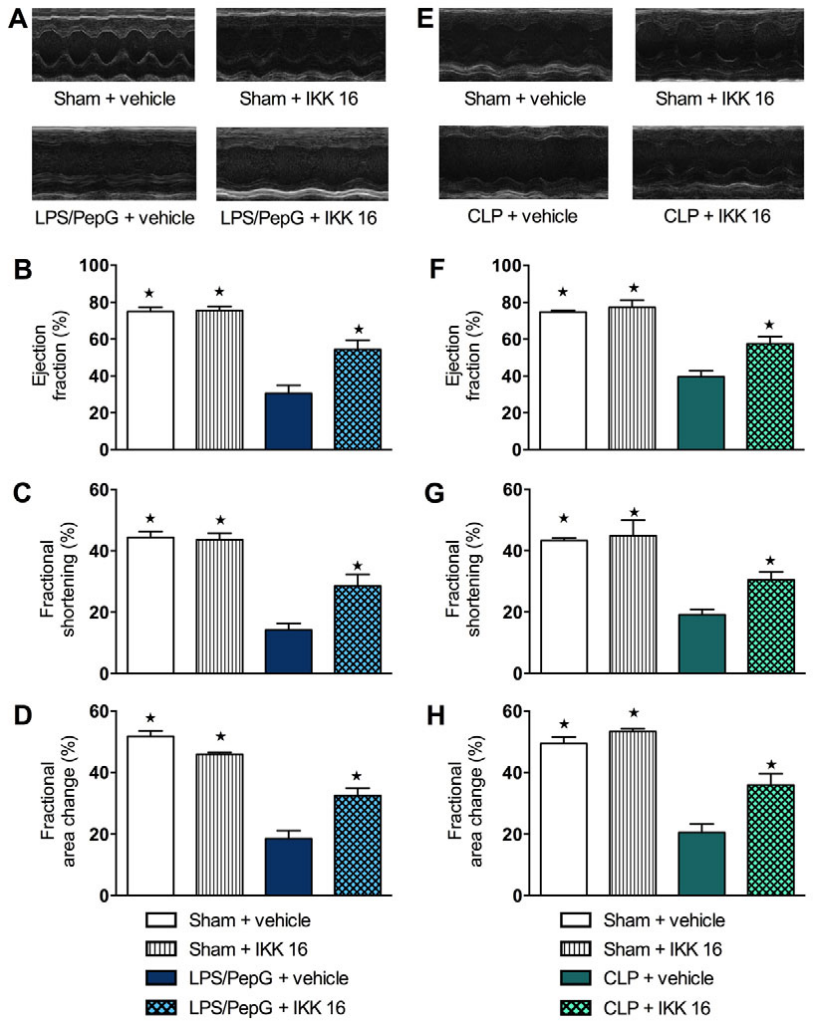
**Effect of IKK 16 on the cardiac dysfunction in mice with LPS/PepG-induced MOD and in septic mice that underwent CLP.** (A-D) Mice received either LPS/PepG or vehicle intraperitoneally. One hour later, mice were either treated with IKK 16 (1 mg/kg body weight i.v.) or vehicle. Cardiac function was assessed at 24 hours. (A) Representative M-mode echocardiograms; percentage (%) (B) ejection fraction (EF), (C) fractional shortening (FS) and (D) fractional area of change (FAC). The following groups were studied: sham + vehicle (*n*=10); sham + IKK 16 (*n*=3); LPS/PepG + vehicle (*n*=9); LPS/PepG + IKK 16 (*n*=10). (E-H) At 1 hour after CLP or sham operation, mice were either treated with IKK 16 (1 mg/kg body weight i.v.) or vehicle. Cardiac function was assessed at 24 hours. (E) Representative M-mode echocardiograms; % (F) EF, (G) FS and (H) FAC. The following groups were studied: sham + vehicle (*n*=7); sham + IKK 16 (*n*=3); CLP + vehicle (*n*=12); CLP + IKK 16 (*n*=12). (A-H) Data are expressed as means ± s.e.m. for *n* number of observations. *P<0.05 versus corresponding control group (LPS/PepG + vehicle or CLP + vehicle).

Left ventricular (LV) function was assessed by echocardiography 24 hours after administration of vehicle or LPS/PepG. There were no significant differences in heart rate or temperature of all groups (*P*>0.05; Table 1). Fig. 1A shows representative M-mode echocardiograms of the studied groups: sham + vehicle, sham + IKK 16, LPS/PepG + vehicle, and LPS/PepG + IKK 16. When compared with sham + vehicle mice, sham mice treated with IKK 16 demonstrated no significant alterations in percentage ejection fraction (EF), fractional shortening (FS) and fractional area of change (FAC) (*P*>0.05; Fig. 1B–D). When compared with sham mice, mice subjected for 24 hours to LPS/PepG demonstrated a significant reduction in percentage EF, FS and FAC (*P*<0.05; Fig. 1B–D), indicating impairment in systolic contractility *in vivo*. Delayed administration of IKK 16 at 1 hour after LPS/PepG significantly attenuated the impairment in systolic contractility associated with LPS/PepG (*P*<0.05; Fig. 1B–D).

**Table 1.**
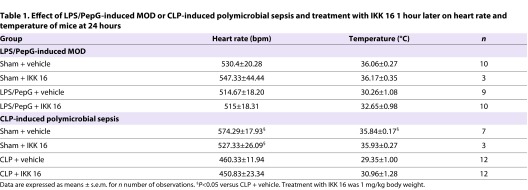
Effect of LPS/PepG-induced MOD or CLP-induced polymicrobial sepsis and treatment with IKK 16 1 hour later on heart rate and temperature of mice at 24 hours

### Effect of CLP-induced polymicrobial sepsis and treatment with IKK 16 on cardiac function in mice

We sought to confirm our findings in a clinically relevant model of polymicrobial sepsis caused by CLP in aged mice. Mice that underwent CLP had a lower body temperature (sham + vehicle versus CLP + vehicle; *P*<0.05) and a lower heart rate (sham + vehicle/sham + IKK 16 versus CLP + vehicle; *P*<0.05) in comparison with sham-operated animals. Fig. 1E shows representative M-mode echocardiograms of the studied groups: sham + vehicle, sham + IKK 16, CLP + vehicle, and CLP + IKK 16. When compared with sham + vehicle mice, sham mice treated with IKK 16 demonstrated no significant alterations in percentage EF, FS and FAC (*P*>0.05; Fig. 1F–H). When compared with sham mice, mice that underwent CLP developed a significant reduction in percentage EF, FS and FAC (*P*<0.05; Fig. 1F–H) at 24 hours, indicating impairment in systolic contractility *in vivo*. Delayed administration of IKK 16 at 1 hour after CLP significantly attenuated the impairment in systolic contractility associated with polymicrobial sepsis (*P*<0.05; Fig. 1F–H).

### Effect of LPS/PepG administration and treatment with IKK 16 on renal dysfunction, hepatocellular injury and lung inflammation

**Fig. 2.**
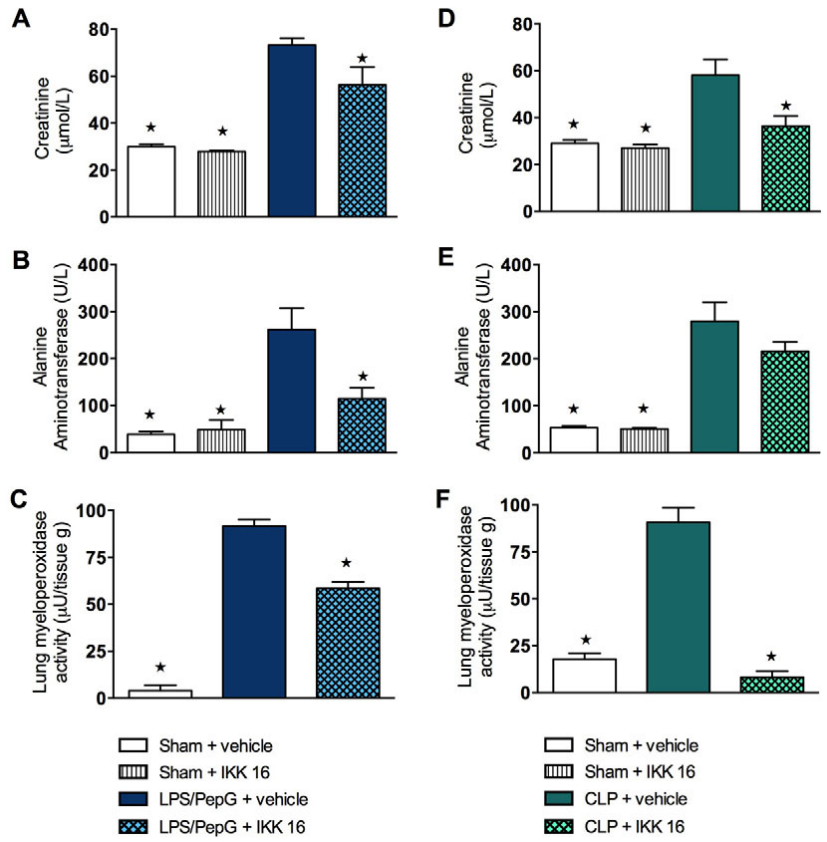
**Effect of IKK 16 on kidney dysfunction, hepatocellular injury and lung inflammation in mice with LPS/PepG-induced MOD and in septic mice that underwent CLP.** (A–C) Mice received either LPS/PepG or vehicle intraperitoneally. One hour later, mice were treated with either IKK 16 (1 mg/kg body weight i.v.) or vehicle. Markers of organ function and/or injury were assessed at 24 hours. The following groups were studied: sham + vehicle (*n*=10); sham + IKK 16 (*n*=3); LPS/PepG + vehicle (*n*=9); LPS/PepG + IKK 16 (*n*=10). (A) Serum creatinine levels; (B) serum alanine aminotransferase (ALT) levels; and (C) myeloperoxidase (MPO) activity in lung tissue. (D–F) At 1 hour after CLP or sham operation, mice were treated with either IKK 16 (1 mg/kg i.v.) or vehicle. Markers of organ function and/or injury were assessed at 24 hours. The following groups were studied: sham + vehicle (*n*=7); sham + IKK 16 (*n*=3); CLP + vehicle (*n*=12); CLP + IKK 16 (*n*=12). (D) Serum creatinine levels; (E) serum ALT levels; and (F) MPO activity in lung tissue. (A–F) Data are expressed as means ± s.e.m. for *n* number of observations. *P<0.05 versus corresponding control group (LPS/PepG + vehicle or CLP + vehicle).

When compared with sham mice, sham mice treated with IKK 16 demonstrated no significant alterations (*P*>0.05) in serum creatinine (Fig. 2A), urea (data not shown), alanine aminotransferase (ALT; Fig. 2B) and aspartate aminotransferase (AST; data not shown). When compared with sham-operated mice, mice subjected to LPS/PepG and treated with vehicle developed, at 24 hours, significant increases (*P*<0.05) in serum creatinine (Fig. 2A), urea (data not shown), ALT (Fig. 2B), AST (data not shown) and lung myeloperoxidase (MPO) activity (Fig. 2C), indicating the development of renal dysfunction (creatinine and urea), hepatocellular injury (ALT and AST) and increased neutrophil infiltration in the lung (MPO). Treatment of mice with IKK 16 at 1 hour after administration of LPS/PepG significantly attenuated the rises (*P*<0.05) in serum creatinine (Fig. 2A), ALT (Fig. 2B), AST (data not shown) and MPO activity in the lung (Fig. 2C). Thus, treatment of mice with IKK 16 attenuated the LPS/PepG-induced MOD that was seen at 24 hours.

### Effect of CLP-induced polymicrobial sepsis and treatment with IKK 16 on renal dysfunction, hepatocellular injury and lung inflammation

We were also able to confirm the above effects of IKK 16 in a model of CLP-induced polymicrobial sepsis 24 hours after surgery. When compared with sham-operated mice, sham mice treated with IKK 16 demonstrated no significant alterations (*P*>0.05) in serum creatinine (Fig. 2A), urea (data not shown), ALT (Fig. 2B) and AST (data not shown). When compared with sham-operated mice, mice subjected to CLP and treated with vehicle developed, at 24 hours, significant increases (*P*<0.05) in serum creatinine (Fig. 2D), urea (data not shown), ALT (Fig. 2E), AST (data not shown) and MPO activity (Fig. 2F), indicating the development of renal dysfunction (creatinine and urea), liver injury (ALT and AST) and increased neutrophil infiltration in the lung (MPO). Treatment of septic mice with IKK 16 at 1 hour after surgery significantly attenuated (*P*<0.05) the rises in serum creatinine (Fig. 2D) and lung MPO activity (Fig. 2F), and ameliorated the rise in ALT (Fig. 2E, *P*>0.05). Thus, treatment of septic mice with IKK 16 attenuated the sepsis-induced MOD.

### Effect of LPS/PepG administration and treatment with IKK 16 on the phosphorylation of IκBα, the nuclear translocation of p65 NF-κB and the iNOS expression in mouse heart tissue

**Fig. 3.**
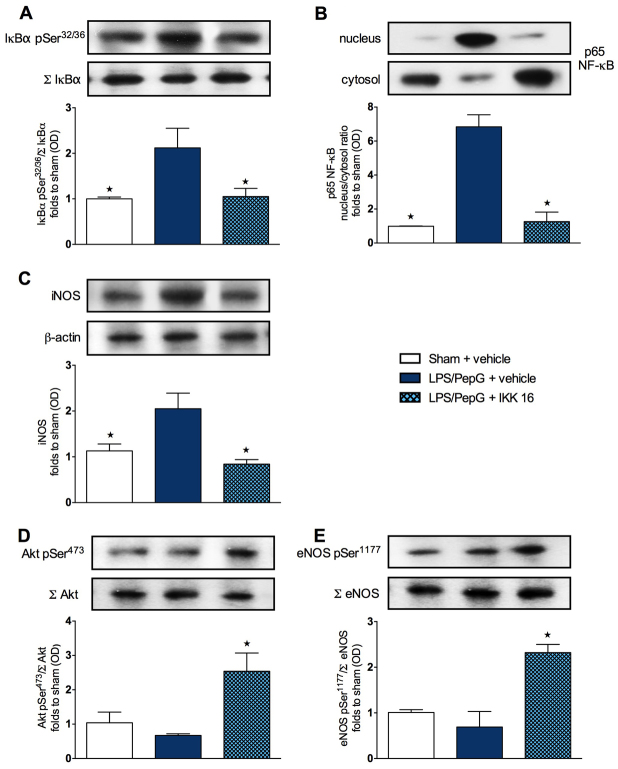
**Effect of IKK 16 on signaling pathways in the hearts of mice with LPS/PepG-induced MOD.** Mice received either LPS/PepG or vehicle. One hour later, mice were treated with either IKK 16 (1 mg/kg body weight i.v.) or vehicle. Signaling events in heart tissue were assessed at 24 hours. Each immunoblot (A–E) is from a single experiment and is representative of three separate experiments. Data are expressed as means ± s.e.m. for *n* number of observations. *P<0.05 versus LPS/PepG + vehicle. All values were corrected for the corresponding β-actin band. Densitometric analysis of the bands is expressed as relative optical density (OD) of (A) phosphorylated IκBα (pSer32/36) corrected for the corresponding total IκBα content (Σ IκBα) and normalized using the related sham band; (B) NFĸB p65 subunit levels in both cytosolic and nuclear fractions expressed as a nucleus:cytosol ratio normalized using the related sham bands; (C) iNOS expression corrected for the corresponding β-actin band; (D) phosphorylated Akt (pSer473) corrected for the corresponding total Akt content (Σ Akt) and normalized using the related sham band; (E) phosphorylated eNOS (pSer1177) corrected for the corresponding total eNOS content (Σ eNOS) and normalized using the related sham band.

To gain a better insight into the potential mechanism(s) underlying the observed beneficial effects of IKK 16 on the sepsis-associated cardiac dysfunction, we investigated the effects of this compound at 24 hours on cell signaling pathways in the hearts of mice subjected to LPS/PepG. When compared with heart tissue at 24 hours from sham mice treated with saline, that from mice subjected to LPS/PepG showed a significant increase (*P*<0.05) in the degree of phosphorylation of IκBα on Ser32/36 (Fig. 3A) and the subsequent nuclear translocation of the NF-κB subunit p65 (Fig. 3B), as well as a significant increase (*P*<0.05) in iNOS expression (Fig. 3C). Treatment of mice subjected to LPS/PepG with IKK 16, however, resulted in a significant attenuation (*P*<0.05) of the increased degree of phosphorylation of IκBα (Fig. 3A), of the increased nuclear translocation of p65 (Fig. 3B) and of the increased iNOS expression (Fig. 3C) in the heart.

### Effect of LPS/PepG administration and treatment with IKK 16 on the phosphorylation of Akt and eNOS in mouse heart tissue

When compared with sham mice treated with saline, mice subjected to LPS/PepG demonstrated no changes (*P*>0.05) in the degree of phosphorylation of Akt on Ser473 (Fig. 3D) and eNOS on Ser1177 (Fig. 3E) in heart tissues. However, treatment of mice subjected to LPS/PepG with IKK 16 resulted in a significantly increased degree of phosphorylation (*P*<0.05) of serine residues on Akt (Fig. 3D) and eNOS (Fig. 3E) in the heart.

### Effect of LPS/PepG administration and treatment with IKK 16 on the phosphorylation of IκBα, nuclear translocation of p65 NF-κB and iNOS expression in mouse liver tissue

**Fig. 4.**
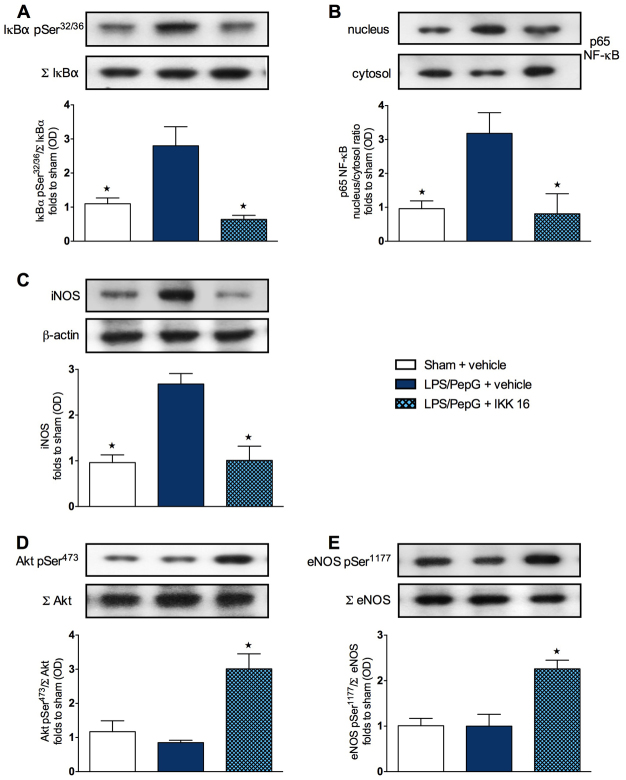
**Effect of IKK 16 on signaling pathways in the livers of mice with LPS/PepG-induced MOD.** Mice received either LPS/PepG or vehicle. One hour later, mice were treated with either IKK 16 (1 mg/kg body weight i.v.) or vehicle. Signaling events in liver tissue were assessed at 24 hours. Each immunoblot (A–E) is from a single experiment and is representative of three separate experiments. Data are expressed as means ± s.e.m. for *n* number of observations. *P<0.05 versus LPS/PepG + vehicle. All values were corrected for the corresponding β-actin band. Densitometric analysis of the bands is expressed as relative optical density (OD) of (A) phosphorylated IκBα (pSer32/36) corrected for the corresponding total IκBα content (Σ IκBα) and normalized using the related sham band; (B) NFĸB p65 subunit levels in both the cytosolic and nuclear fractions expressed as a nucleus:cytosol ratio normalized using the related sham bands; (C) iNOS expression corrected for the corresponding β-actin band; (D) phosphorylated Akt (pSer473) corrected for the corresponding total Akt content (Σ Akt) and normalized using the related sham band; (E) phosphorylated eNOS (pSer1177) corrected for the corresponding total eNOS content (Σ eNOS) and normalized using the related sham band.

We sought to elucidate the above-described signaling events in the liver. When compared with sham mice treated with saline, mice subjected to LPS/PepG showed a significant increase (*P*<0.05) in the phosphorylation of IκBα on Ser32/36 (Fig. 4A), the nuclear translocation of the NF-κB subunit p65 (Fig. 4B) and iNOS expression (Fig. 4C) in liver tissues at 24 hours. Treatment of mice subjected to LPS/PepG with IKK 16, however, resulted in a significant attenuation (*P*<0.05) of the increased phosphorylation of IκBα (Fig. 4A), of the increased nuclear translocation of p65 (Fig. 4B) and of the increased iNOS expression (Fig. 4C) in the liver.

### Effect of LPS/PepG administration and treatment with IKK 16 on the phosphorylation of Akt and eNOS in mouse liver tissue

When compared with sham mice treated with saline, mice subjected to LPS/PepG had no changes (*P*>0.05) in the degree of phosphorylation of Akt on Ser473 (Fig. 4D) and eNOS on Ser1177 (Fig. 4E) at 24 hours. However, treatment of mice subjected to LPS/PepG with IKK 16 resulted in a significantly increased degree of phosphorylation (*P*<0.05) of serine residues on Akt (Fig. 4D) and eNOS (Fig. 4E) in the liver.

### Effect of CLP and treatment with IKK 16 on the phosphorylation of IκBα, the nuclear translocation of p65 NF-κB and the iNOS expression in mouse heart tissue

**Fig. 5.**
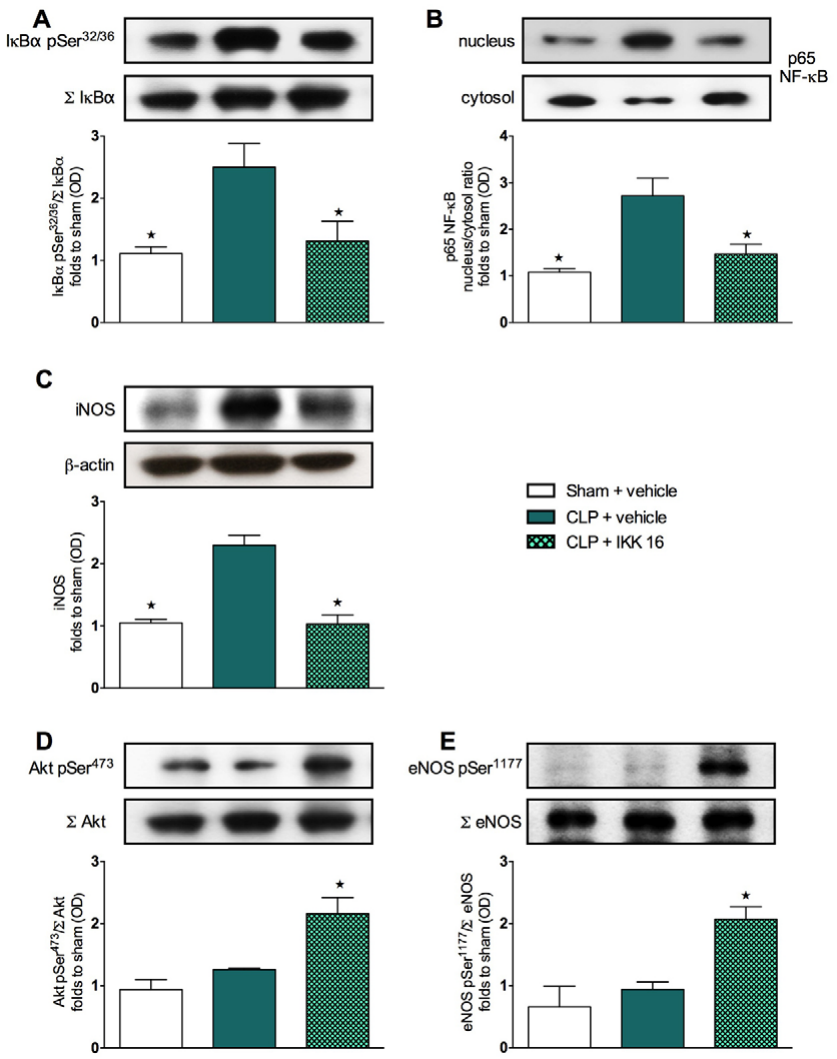
**Effect of IKK 16 on signaling pathways in the hearts of mice with sepsis-induced MOD.** Mice underwent either CLP or sham operation. One hour later, mice were treated with either IKK 16 (1 mg/kg body weight i.v.) or vehicle. Signaling events in heart tissue were assessed at 24 hours. Each immunoblot (A–E) is from a single experiment and is representative of three separate experiments. Data are expressed as means ± s.e.m. for *n* number of observations. *P<0.05 versus CLP + vehicle. All values were corrected for the corresponding β-actin band. Densitometric analysis of the bands is expressed as relative optical density (OD) of (A) phosphorylated IκBα (pSer32/36) corrected for the corresponding total IκBα content (Σ IκBα) and normalized using the related sham band; (B) NFĸB p65 subunit levels in both the cytosolic and nuclear fractions expressed as a nucleus:cytosol ratio normalized using the related sham bands; (C) iNOS expression corrected for the corresponding β-actin band; (D) phosphorylated Akt (pSer473) corrected for the corresponding total Akt content (Σ Akt) and normalized using the related sham band; (E) phosphorylated eNOS (pSer1177) corrected for the corresponding total eNOS content (Σ eNOS) and normalized using the related sham band.

We sought to confirm the signaling events described above for the model of LPS/PepG-induced MOD in the model of CLP-induced polymicrobial sepsis to further elucidate the potential mechanism(s) underlying the observed beneficial effects of IKK 16 on the sepsis-associated cardiac dysfunction. Therefore, we first investigated the effects of this compound on cell signaling pathways in the hearts of mice that underwent CLP at 24 hours. When compared with sham mice treated with saline, mice that underwent CLP demonstrated a significant increase in the degree of phosphorylation (*P*<0.05) of IκBα on Ser32/36 (Fig. 5A) and the subsequent nuclear translocation of the NF-κB subunit p65 (Fig. 5B), as well as a significant increase (*P*<0.05) in iNOS expression (Fig. 5C) in heart tissues at 24 hours. Treatment of septic mice with IKK 16, however, resulted in a significant attenuation (*P*<0.05) of the increased degree of phosphorylation of IκBα (Fig. 5A), of the increased nuclear translocation of p65 (Fig. 3B) and of the increased iNOS expression (Fig. 5C) in the heart.

### Effect of CLP and treatment with IKK 16 on the phosphorylation of Akt and eNOS in mouse heart tissue

When compared with sham mice treated with saline, mice that underwent CLP showed no changes (*P*>0.05) in the degree of phosphorylation of Akt on Ser473 (Fig. 5D) and eNOS on Ser1177 (Fig. 5E) in heart tissues. However, treatment of septic mice with IKK 16 resulted in a significantly increased degree of phosphorylation (*P*<0.05) of serine residues on Akt (Fig. 5D) and eNOS (Fig. 5E) in the heart.

### Effect of CLP and treatment with IKK 16 on the phosphorylation of IκBα, nuclear translocation of p65 NF-κB and iNOS expression in mouse liver tissue

**Fig. 6.**
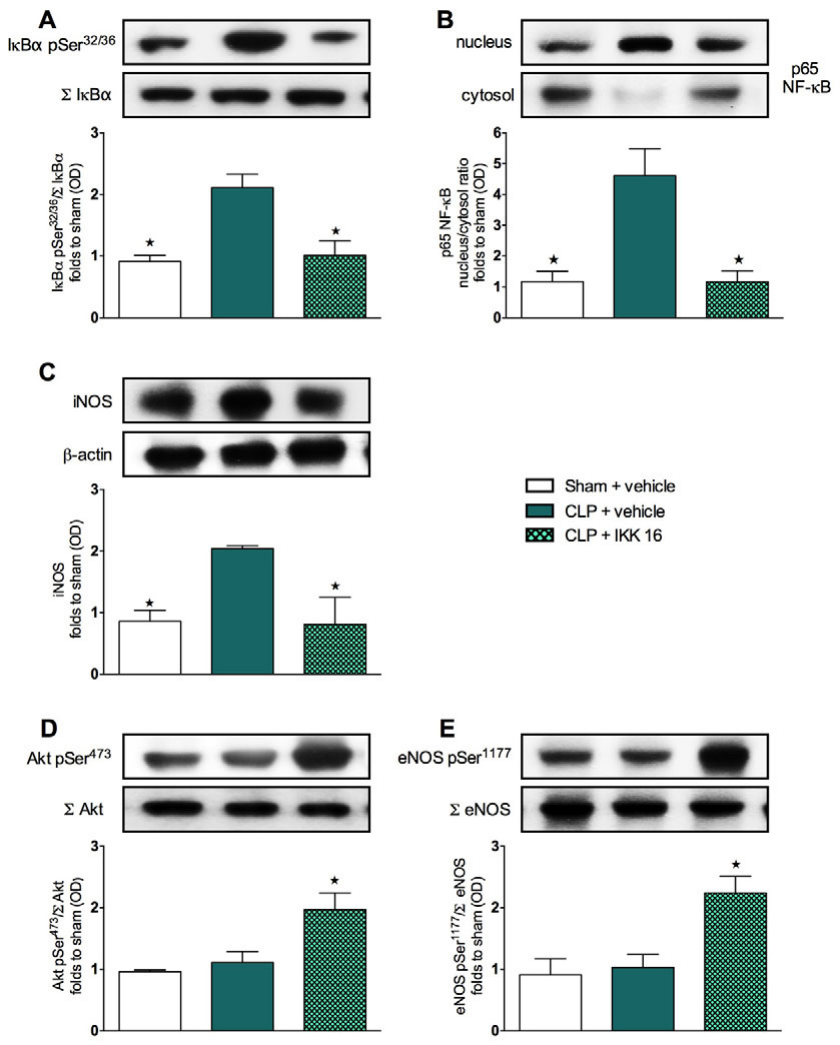
**Effect of IKK 16 on signaling pathways in the livers of mice with sepsis-induced MOD.** Mice underwent either CLP or sham operation. One hour later, mice were treated with either IKK 16 (1 mg/kg body weight i.v.) or vehicle. Signaling events in liver tissue were assessed at 24 hours. Each immunoblot (A–E) is from a single experiment and is representative of three separate experiments. Data are expressed as means ± s.e.m. for *n* number of observations. *P<0.05 versus CLP + vehicle. All values were corrected for the corresponding β-actin band. Densitometric analysis of the bands is expressed as relative optical density (OD) of (A) phosphorylated IκBα (pSer32/36) corrected for the corresponding total IκBα content (Σ IκBα) and normalized using the related sham band; (B) NFĸB p65 subunit levels in both the cytosolic and nuclear fractions expressed as a nucleus:cytosol ratio normalized using the related sham bands; (C) iNOS expression corrected for the corresponding β-actin band; (D) phosphorylated Akt (pSer473) corrected for the corresponding total Akt content (Σ Akt) and normalized using the related sham band; (E) phosphorylated eNOS (pSer1177) corrected for the corresponding total eNOS content (Σ eNOS) and normalized using the related sham band.

We then investigated the effects at 24 hours of IKK 16 on cell signaling pathways in the livers of mice that underwent CLP. When compared with sham mice treated with saline, mice that underwent CLP demonstrated a significant increase (*P*<0.05) in the phosphorylation of IκBα on Ser32/36 (Fig. 6A), the nuclear translocation of the NF-κB subunit p65 (Fig. 6B) and iNOS expression (Fig. 6C) in liver tissues at 24 hours. Treatment of septic mice with IKK 16, however, resulted in a significant attenuation (*P*<0.05) of the increased phosphorylation of IκBα (Fig. 6A), of the increased nuclear translocation of p65 (Fig. 6B) and of the increased iNOS expression (Fig. 6C) in the liver.

### Effect of CLP and treatment with IKK 16 on the phosphorylation of Akt and eNOS in mouse liver tissue

When compared with sham mice treated with saline, mice that underwent CLP showed no changes in the degree of phosphorylation (*P*>0.05) of Akt on Ser473 (Fig. 6D) and eNOS on Ser1177 (Fig. 6E) at 24 hours. However, treatment of septic mice with IKK 16 resulted in a significantly increased degree of phosphorylation (*P*<0.05) of serine residues on Akt (Fig. 6D) and eNOS (Fig. 6E) in the liver.

## DISCUSSION

The pathophysiology of sepsis is yet to be fully understood; however, current understanding of the development of sepsis-induced MOD highlights the involvement of various cell populations and cell signaling pathways in the onset of this condition, leading to a systemic, sometimes excessive, immune response to an infection. There is good evidence that increased activation of the transcription factor NF-κB with a subsequent regulation of pro-inflammatory mediators plays a crucial role in the development of organ dysfunction occurring in sepsis (Abraham and Singer, 2007). Clinical studies suggest a correlation between enhanced NF-κB activation in sepsis and poor outcome (Arnalich et al., 2000; Yang et al., 2003). Senftleben and Karin reasoned as early as in 2002 that the IKK–NF-κB pathway might play an *‘*exceptionally important role due to the rapidity of activation and its unique regulation’ in critical diseases (Senftleben and Karin, 2002). The current study was designed to elucidate the role of the selective inhibition of the IKK complex *in vivo* in two different mouse models of experimental sepsis.

We first developed a model of severe LPS/PepG-induced MOD in young mice. Our results demonstrate that co-administration of the Gram-negative (LPS) and Gram-positive (PepG) bacterial cell wall components caused a substantial reduction in percentage EF, FS and FAC as well as significant increases in serum creatinine, urea, ALT and AST, and in lung MPO activity (marker for neutrophil accumulation in the lung), indicating an impairment in systolic contractility as well as the development of renal dysfunction, hepatocellular injury and lung inflammation and, hence, MOD. Our results are in line with other studies, which have described that the LPS/PepG synergism causes the release of inflammatory mediators and MOD in rats (Dugo et al., 2005; Dugo et al., 2004; Wray et al., 2001).

Although sepsis can occur in previously healthy and/or young individuals, the elderly, surgical patients and patients with chronic diseases are predisposed to suffer from this condition (De Gaudio et al., 2009; Girard et al., 2005). Therefore, we wished to strengthen our study by confirming our observations in a clinically relevant model of severe polymicrobial sepsis with antibiotic therapy and fluid-resuscitation caused by CLP in aged mice, which we have described recently (Coldewey et al., 2013; Khan et al., 2013).

The IKK inhibitor IKK 16 used in this study was designed and first synthesized by Waelchli et al. (Waelchli et al., 2006). We decided to use a treatment protocol that could theoretically be applicable in a clinical setting in septic patients with a relatively low dose of IKK 16, an administration time after induction of disease and an intravenous application route.

We report here, for the first time, that the selective delayed inhibition of IKK with a low dose of IKK 16 attenuates the cardiac dysfunction, renal dysfunction, hepatocellular injury and lung inflammation caused by co-administration of LPS/PepG in young mice or CLP-induced polymicrobial sepsis in aged mice.

It should be noted that we observed a similar magnitude of organ dysfunction and injury in both models, although mice that underwent CLP had on average a lower body temperature and heart rate than LPS/PepG-challenged mice. However, the effect of IKK 16 on the MPO activity in the lung, as a marker for neutrophil infiltration, was much greater in the CLP model than in the LPS/PepG model, whereas the reduction of ALT and AST by IKK 16 was not significant in the CLP model, but was in the model of LPS/PepG.

We can only speculate which different variables in our models lead to the observed distinct treatment effects of IKK 16. Most likely, the liver might be affected more severely in the model of sepsis owing to its localization close to the continuous infectious stimulus in the peritoneal cavity, subsequently leading to a less pronounced beneficial effect of IKK 16 on hepatocellular injury than in the model of LPS/PepG.

In addition to kidney and liver dysfunction, cardiac dysfunction is a common clinical complication of severe sepsis and septic shock (Flynn et al., 2010) and is known to worsen prognosis (Celes et al., 2013). Echocardiographic studies suggest that myocardial depression occurs in 40–50% of patients with prolonged septic shock (Rudiger and Singer, 2007). Contractile dysfunction in the septic heart is clinically characterized as biventricular dilatation, reversible decrease in EF, diminished blood pressure response to intravenous fluids and blunted ability to augment cardiac output despite increased levels of circulating catecholamines (Flynn et al., 2010), and is fully reversible in survivors. To date, the principal mechanisms proposed for intrinsic myocardial depression during sepsis support a considerable role for functional rather than anatomical abnormalities or cell death (Celes et al., 2013; Rudiger and Singer, 2007).

We sought to elucidate underlying signaling mechanism(s) for the beneficial effects of IKK 16. In hearts and livers of mice that were subjected to LPS/PepG or underwent CLP, we observed increased phosphorylation of IκBα on Ser32/36, increased nuclear translocation of the NF-κB subunit p65 and increased iNOS expression, all of which were abolished following the treatment with IKK 16 at 1 hour after co-administration of LPS/PepG or CLP. Our results indicate that the delayed administration of IKK 16 sufficiently inhibits IKK, resulting in the decreased phosphorylation and subsequently less degradation of IκBs, which in turn leads to less activation of NF-κB via sequestration of NF-κB in an inactive state in the cytoplasm. Subsequently, NF-κB-dependent proteins are less expressed, e.g. iNOS (which plays a crucial role in the hypotension and MOD associated with septic shock) (Barth et al., 2006; Brady et al., 1992; Kengatharan et al., 1996; Szabó et al., 1994; Thiemermann and Vane, 1990).

As a likely consequence, this would also affect various cytokines, which are known to be expressed in an NF-κB-dependent manner and to play an important role in the pathophysiology of sepsis. This assumption is strengthened by a study of Waelchli et al. that has shown the efficacy of the compound to inhibit TNFα release into the plasma upon LPS challenge in the rat (Waelchli et al., 2006). In their study, IKK 16 was administered subcutaneously (30 mg/kg body weight) or orally (30 mg/kg body weight) 1 hour prior to LPS challenge. At 4 hours after the challenge, both routes of administration resulted in a significant reduction of TNFα of 86% (subcutaneously) and 75% (orally) (Waelchli et al., 2006).

The role of NO in septic shock is still controversial (Laubach et al., 1998; Laubach et al., 1995; MacMicking et al., 1995; Wei et al., 1995). For example, the mortality of iNOS-deficient mice in septic shock is not altered or it is increased (Laubach et al., 1998; Laubach et al., 1995; Mao et al., 2013). These findings are in line with clinical studies showing that treatment with the non-selective nitric oxide synthase (NOS) inhibitor 546C88 does not improve the outcome of septic patients (Bakker et al., 2004; López et al., 2004; Watson et al., 2004). Our current hypothesis that inhibition of iNOS expression (here secondary to inhibition of NF-κB activation by IKK 16) importantly contributes to the organ protection in severe sepsis is supported by the following findings: (1) iNOS-deficient mice show reduced non-specific inflammatory response and have a lower mortality rate than wild-type mice (MacMicking et al., 1995; Wei et al., 1995); (2) inhibition of NO synthesis reduces the LPS-induced hypotension in the rat (Thiemermann and Vane, 1990); (3) NO-mediated hyporeactivity to noradrenaline precedes the induction of iNOS in endotoxic shock (Szabó et al., 1993); (4) iNOS inhibitors exert beneficial effects and improve survival in rodent models of septic shock (Szabó et al., 1994); (5) enhanced formation of NO by iNOS importantly contributes to the circulatory failure, hepatocellular injury, respiratory dysfunction and metabolic acidosis, but not the renal failure, caused by lipoteichoic acid/PepG in the rat (Kengatharan et al., 1996); and (6) enhanced formation of NO by iNOS contributes to the cardiac dysfunction associated with septic shock (Brady et al., 1992; Barth et al., 2006).

Furthermore, our results demonstrate that treatment with IKK 16 also has a large impact on the regulation of the well-described Akt-eNOS survival pathway. One may consider this result to be quite unexpected given that Akt-eNOS are upstream of IKK. However, there is evidence to suggest that heat shock protein 90 (Hsp90) binds directly with the kinase domains of IKKα and IKKβ (Chen et al., 2002), and is also a key molecular chaperone protein for eNOS regulation (Fleming and Busse, 2003). The interaction of Hsp90 and eNOS creates a complex with Akt, which allows eNOS and Akt to both function on the same domain of Hsp90 (Fontana et al., 2002). This interaction is increased when IKK is inhibited, resulting in increased NO production (Mohan et al., 2009).

Akt is a member of the phosphoinositide-3-kinase signal transduction enzyme family. When phosphorylated by its upstream regulator (phosphoinositide-dependent kinase), Akt regulates cellular activation, inflammatory responses, chemotaxis and apoptosis, and thus modulates cell survival and growth (Cantley, 2002). We document here that co-administration of LPS/PepG or sepsis caused no changes in the phosphorylation of Akt on Ser473 in heart and liver tissues, but treatment of mice with IKK 16 at 1 hour after co-administration of LPS/PepG or CLP resulted in a significantly increased phosphorylation of Akt, and, hence, activation of Akt. It is known that activation of Akt results in the phosphorylation of eNOS on Ser1177, which in turn causes activation of eNOS, resulting in an enhanced formation of NO in the microcirculation. In sepsis, activation of eNOS is seen to be beneficial, because the enhanced formation of NO in the microcirculatory compartment (e.g. in the heart and liver) causes local vasodilation, inhibits adhesion of platelets and neutrophils, and regulates angiogenesis (Khan et al., 2010; Tyml, 2011). In this study, co-administration of LPS/PepG or CLP caused no changes in the phosphorylation of eNOS on Ser1177 in heart and liver tissues. Treatment of mice with IKK 16 at 1 hour after co-administration of LPS/PepG or CLP, however, resulted in a significantly increased phosphorylation of eNOS. Thus, activation of eNOS (possibly secondary to activation of Akt) might contribute to the beneficial effects of IKK 16 reported here.

## Conclusion

The activation of NF-κB contributes to the organ dysfunction and/or injury in experimental sepsis caused by either excessive inflammation via co-administration of LPS/PepG in young mice or polymicrobial sepsis following CLP in aged mice. Most notably, we were able to convincingly demonstrate, for the first time, that the delayed selective inhibition of IKK reduces the MOD associated with sepsis. Our results suggest that the protective effect of IKK 16 in experimental sepsis is (at least in part) attributable to an anti-inflammatory and endothelial protective effect mediated by inhibition of inflammation through NF-κB, subsequent decreased iNOS expression and interestingly the activation of the well-known Akt-eNOS survival pathway. We propose that selective inhibition of IKK might be a novel therapeutic strategy for the prevention or therapy of the MOD associated with sepsis and other conditions associated with systemic inflammation.

## MATERIALS AND METHODS

### Animals

The animal protocols followed in this study were approved by the local ‘Animal Use and Care Committee’ in accordance with the derivatives of both the ‘Home Office Guidance on the Operation of the Animals (Scientific Procedures) Act 1986’, published by Her Majesty’s Stationery Office and the ‘Guide for the Care and Use of Laboratory Animals’ of the National Research Council. This study was carried out on 32 2-month-old male C57BL/6 mice, weighing 20–30 g, and 34 8-month-old male C57BL/6 mice (Charles River Laboratories UK Ltd, Kent, UK), weighing 35–50 g, receiving a standard diet and water *ad libitum*.

### Quantification of organ dysfunction and/or injury

Organ dysfunction and/or injury was assessed in mice subjected to LPS/PepG or CLP at 24 hours. Mice were anesthetized with 1.5 ml/kg body weight i.p. of a ketamine (100 mg/ml)/xylazine (20 mg/ml) solution in a 2:1 ratio before being sacrificed. Approximately 0.7 ml of blood was collected by cardiac puncture into non-heparinized syringes and immediately decanted into serum gel S/1.3 tubes (Sarstedt, Nürnbrecht, Germany), after which the heart was removed to terminate the experiment. The samples were centrifuged at 9900 ***g*** for 5 minutes to separate serum, which was sent to an independent laboratory (IDEXX Laboratories, Buckinghamshire, UK) for analysis of serum creatinine, urea, AST and ALT. Additionally, organ samples were taken, snap frozen and stored at −80°C for further analyses.

### Experimental design

#### Model of LPS/PepG-induced MOD

Two-month-old male C57BL/6 mice received LPS (9 mg/kg body weight) and PepG (3 mg/kg body weight) in 0.9% saline (5 ml/kg body weight) intraperitoneally. Sham mice were not subjected to LPS/PepG, but were otherwise treated the same way. At 1 hour after LPS/PepG co-administration, mice were treated either with IKK 16 (1 mg/kg body weight i.v.) or vehicle (5 ml/kg body weight 10% DMSO i.v.). At 24 hours the experiment was terminated and organ and blood samples were collected for quantification of organ dysfunction and/or injury. Mice were randomly allocated into four different groups: (1) sham + vehicle (*n*=10); (2) sham + IKK 16 (*n*=3); (3) LPS/PepG + vehicle (*n*=9); (4) LPS/PepG + IKK 16 (*n*=10).

#### Model of polymicrobial sepsis caused by CLP

Eight-month-old male C57BL/6 mice were subjected to CLP. Sham mice were not subjected to CLP but were otherwise treated the same way. We followed the original CLP protocol introduced by Wichterman and co-workers (Wichterman et al., 1980) with slight modifications including analgesia, antibiotic therapy and fluid resuscitation as described previously (Coldewey et al., 2013; Khan et al., 2013). An 18-G needle was used with the double puncture technique in order to generate reproducible MOD during the early phase of sepsis in aged mice. Briefly, mice were anesthetized [1.5 ml/kg body weight of a ketamine (100 mg/ml)/xylazine (20 mg/ml) solution in a 2:1 ratio i.p.]. Buprenorphine (0.05 mg/kg body weight i.p.) was injected additionally to provide adequate analgesia. The rectal temperature of the animals was maintained at 37°C with a homeothermic blanket. The abdomen was opened via a 1.5 cm midline incision, and the cecum exposed. The cecum was ligated below the ileocecal valve and punctured at both opposite ends. After a small amount of fecal matter was extruded from both ends, the cecum was placed back in its anatomical position and the abdomen was sutured. Ringer’s solution was administered directly after surgery (1 ml/mouse) and 6 hours and 18 hours after surgery (0.5 ml/mouse) for fluid resuscitation. Antibiotic (Imipenem/Cilastin; 20 mg/kg body weight s.c.) and analgesia (buprenorphine; 0.05 mg/kg body weight i.p.) was administered 6 hours and 18 hours after surgery. At 1 hour after CLP, mice were treated either with IKK 16 (1 mg/kg body weight i.v.) or vehicle (5 ml/kg body weight 10% DMSO i.v.). At 24 hours the experiment was terminated and organ and blood samples were collected for quantification of sepsis-induced organ dysfunction and/or injury. Mice were randomly allocated into four different groups: (1) sham + vehicle (*n*=7); (2) sham + IKK 16 (*n*=3); (3) CLP + vehicle (*n*=12); (4) CLP + IKK 16 (*n*=12).

### Assessment of cardiac function *in vivo*

Cardiac function was assessed in mice by echocardiography *in vivo* as reported previously (Kapoor et al., 2010; Khan et al., 2013). At 24 hours after LPS/PepG challenge or after CLP, anesthesia was induced with 3% isoflurane and was maintained at 0.5–0.7% for the duration of the procedure. Before assessment of cardiac function, mice were allowed to stabilize for at least 10 minutes. During echocardiography, the heart rate was obtained from ECG tracing and the temperature was monitored via a rectal probe. Two-dimensional and M-mode echocardiography images were recorded using a Vevo-770 imaging system (VisualSonics, Toronto, Ontario, Canada). Percentage FAC was assessed with a two-dimensional trace and percentage EF and FS were calculated from the M-mode measurements in the parasternal short axis view at the level of the papillary muscles.

### Immunoblot analyses

Semi-quantitative immunoblot analyses were carried out in mouse heart and liver tissues as described previously (Collino et al., 2006). We assessed the degree of phosphorylation of IκBα on Ser32/36, Akt on Ser473 and eNOS on Ser1177, as well as the nuclear translocation of the p65 subunit of NF-κB (nucleus:cytosol ratio) and iNOS expression. Briefly, mouse heart and liver samples were homogenized in 10% homogenization buffer and centrifuged at 1323 ***g*** for 5 minutes at 4°C. Supernatants were removed and centrifuged at 16,215 ***g*** at 4°C for 40 minutes to obtain the cytosolic fraction. The pelleted nuclei were re-suspended in extraction buffer and centrifuged at 16,215 ***g*** for 20 minutes at 4°C. The resulting supernatants containing nuclear proteins were carefully removed, and protein content was determined on both nuclear and cytosolic extracts using a bicinchoninic acid (BCA) protein assay following the manufacturer’s directions (Thermo Fisher Scientific, Runcorn, UK). Proteins were separated by 8% sodium-dodecyl-sulphate–PAGE (SDS-PAGE) and transferred to a polyvinyldenedifluoride (PVDF) membrane, which was then incubated with a primary antibody (mouse anti-total IκBα, dilution 1:1000; mouse anti-IκBα pSer32/36, dilution 1:1000; rabbit anti-NF-κB p65, dilution 1:1000; rabbit anti-total iNOS, dilution 1:200; rabbit anti-total eNOS, dilution 1:200; goat anti-peNOS Ser1177, dilution 1:200; rabbit anti-total Akt, dilution 1:1000; mouse anti-pAkt Ser473, dilution 1:1000). Blots were then incubated with a secondary antibody conjugated with horseradish peroxidase (dilution 1:10,000) for 30 minutes at room temperature and developed with the ECL detection system. The immunoreactive bands were visualized by autoradiography. Densitometric analysis of the bands was performed using the Gel Pro Analyzer 4.5, 2000 software (Media Cybernetics, Silver Spring, MD). Each group was then adjusted against corresponding sham data to establish relative protein expression when compared with sham animals.

### Determination of MPO activity in lung tissue

MPO was extracted from the tissue as described by Barone et al. (Barone et al., 1991) with slight modifications. MPO activity, used as a marker for neutrophil accumulation in tissues, was determined as previously described (Collino et al., 2009).

### Statistics

All values described in the text and figures are presented as mean ± standard error of the mean (s.e.m.) of *n* observations, where *n* represents the number of animals studied. Statistical analysis was performed using GraphPad Prism 6.0b (GraphPad Software, San Diego, CA). Data without repeated measurements were assessed by one-way ANOVA followed by Dunnett’s post-hoc test. A *P*-value of less than 0.05 was considered to be statistically significant.

### Materials

Unless otherwise stated, all compounds in this study were purchased from Sigma-Aldrich Company Ltd (Poole, Dorset, UK). All solutions were prepared using non-pyrogenic saline [0.9% (w/v) NaCl; Baxter Healthcare Ltd, Thetford, Norfolk, UK]. IKK 16 was purchased from Tocris Bioscience (Bristol, UK). Antibodies for immunoblot analysis were purchased from Santa Cruz Biotechnology, Inc. (Heidelberg, Germany).
